# Hair Care and Hair‐Focused Repetitive Behaviors: A Descriptive Cross‐Sectional Study

**DOI:** 10.1002/hsr2.71730

**Published:** 2026-01-20

**Authors:** Linda Hollatz, Alexander L. Gerlach

**Affiliations:** ^1^ Clinical Psychology and Psychotherapy, University of Cologne Cologne Germany

**Keywords:** hair care practices, hair pulling disorder, trichotillomania

## Abstract

**Background and Aims:**

Hair‐Focused Repetitive Behavior Disorders (HFRBDs), commonly associated with trichotillomania (TTM), including hair pulling, manipulation, or ingestion, can cause significant emotional, physical, and social distress. This study aimed to (1) identify hair care‐related behaviors among individuals with HFRBDs; (2) explore how these practices relate to hair‐pulling behaviors; and (3) examine the potential role of hair professionals in supporting affected individuals.

**Methods:**

An online survey was conducted with adult participants (*n* = 195) who self‐identified as having HFRBDs involving scalp hair. The survey assessed hair‐pulling behavior, personal and professional hair care routines, and perceived effects of these practices. Both quantitative and qualitative data were collected and analyzed.

**Results:**

Findings showed that constructive hair care practices often coexisted with hair‐pulling behaviors. Participants reported that washing (54.1%) and haircutting (34.1%) helped reduce pulling urges, while styling (8.7%) and combing/brushing (15.4%) had minimal effect. Notably, 27.7% stated that their own touch increased urges, while 9.8% found that another person's touch reduced them. Thematic analysis of 501 open responses revealed categories such as pre‐pulling routines, sensory responses to hair texture, and varied experiences with hair salons.

**Conclusion:**

The study highlights the complex relationship between hair care and HFRBDs. Tailored hair care strategies may offer meaningful support for individuals with HFRBDs and could be integrated into guidance provided by clinicians, dermatologists, and hair professionals. Personalized interventions may improve overall management of the condition.

Individuals with Trichotillomania (TTM), also called hair‐pulling disorder (HPD), are likely to experience significant physical, emotional, mental, and social distress, such as tension, shame, guilt, and isolation. An estimated 1.7% of the general population is affected by TTM [1]. Hair is pulled from various body areas, including the scalp, eyebrows, eyelids, pubic region, and beard. This behavior may be limited to a small, specific location, such as a spot on the scalp, or involve multiple areas and types of hair. As Christensen (1995) observed, the scalp is the most common site (72.8%), followed by the eyebrows (56.4%) and the pubic region (50.7%). The frequency of hair pulling can range from occasional episodes to several hours daily. The intensity of the behavior also differs, from forcefully grabbing and tearing out multiple hairs at once to gently gliding a finger along a single strand until it loosens and slips out. The visible consequences include bald spots, thinning hair, and skin irritation [2]. Social challenges arise from self‐imposed isolation, feelings of exclusion, conflicts with loved ones [3], and difficulties managing daily life and professional obligations [4]. Since each individual expresses the symptomatic behavior uniquely, the disorder is considered highly heterogeneous [5, 6, 7].

Current interventions for TTM are primarily based on psychological models, with evidence‐based treatments centered on cognitive‐behavioral therapies, such as Habit Reversal Training (HRT), Dialectical Behavior Therapy (DBT), and Acceptance and Commitment Therapy (ACT). While these approaches focus on underlying cognitive, emotional, or behavioral mechanisms, the present study, in contrast, investigates more practical, hair‐care–oriented options that may complement existing models by targeting everyday routines, self‐perception, and sensory experiences related to hair.

## 1 Terminology, Classification, and Variations

The *International Classification of Diseases* Eleventh Revision (ICD‐11 [8]); and the *Diagnostic and Statistical Manual of Mental Disorders*, Fifth Edition (DSM‐5 [9]); positioned TTM and Excoriation (Skin Picking) Disorder as Body‐Focused Repetitive Behavior Disorders (BFRBDs) under the Obsessive‐Compulsive and Related Disorders category. While TTM and Excoriation Disorder are not explicitly grouped as BFRBDs in the DSM‐5, the accompanying text acknowledges that both disorders share the characteristic of repetitive body‐focused behaviors.

TTM and related hair‐focused repetitive behaviors vary in manifestation, intensity, and frequency, for example, regarding micro‐actions and tools, such as the thumb and index finger of one or both hands, fingernails, tweezers, scissors, or even teeth [10]. Differences also exist in the phases before and after hair pulling. For example, pulling may occur abruptly or be preceded by scanning, visually inspecting, or feeling for specific hairs. Once a hair is pulled, individuals may drop it, examine it, engage in intentional collection and disposal, or bring parts of it, like the root or shaft, near the lips or mouth, or even chew or swallow the hair [11].

BFRBDs, also known as pathological grooming disorders, encompass a wide range of behaviors that focus on specific body parts, including hair, skin, nails, eyes, ears, nose, and mouth. While “grooming” typically refers to everyday self‐care practices, such as brushing hair, that improve appearance, in clinical settings, it describes compulsive actions that impact a person's well‐being. In an extensive online community survey (*N* = 2705), Maraz et al. [12] identified pathological grooming as a common element among trichotillomania (TTM), skin picking disorder (SPD), and nail‐biting disorder (NBD). The term has also been used in animal studies of BFRBDs, including research on mice, birds, and dogs [13, 14]. However, because of its broader and sometimes problematic implications—including its use to describe predatory behavior—the term *“grooming”* is replaced with *“body care”* in this article to remain neutral.

Importantly, not all BFRBDs appear to serve any body care function. Examples include Cheilitis factitia, Dermatophagia, thumb sucking, joint cracking, and various oral‐focused behaviors (e.g., Morsicatio buccarum, Trichophagia). Differentiating between BFRBDs that stem from or escalate into pathological body care routines versus those with no apparent body care origin may offer a more refined understanding of the spectrum of these disorders. Throughout this text, we will use the term *Hair‐Focused Repetitive Behaviors (HFRBDs)* to encompass all variations of hair‐related body‐focused repetitive behaviors. This includes, but is not limited to TTM, trichoteiromania, and trichotemnomania. This distinction is particularly relevant for the current study, which focuses on behaviors that may initially resemble benign self‐care practices but can develop into clinically impairing patterns.

The destructive actions individuals inflict on their hair—and, by extension, on themselves—naturally draw the primary attention of affected individuals, researchers, and clinicians. However, this study focuses on the opposing constructive behaviors that an affected individual engages in to care for their hair, including daily care, cutting, styling, and attending to wounded parts. The potential of hair care as a form of self‐care, both when practiced by affected individuals and supported by the complementary, informed, and empathetic guidance of hair professionals, remains largely unexplored. This prompted the central questions of this study: Do affected individuals engage in constructive hair care, and does such care not only address the effects of HFRBDs but also help prevent, redirect, or even eliminate them?

## 2 Literature Review

Research on TTM and its intersection with hair care has yielded only a few references and one study. These addressed three main topics: “self‐care and hair care” [15], “positive experiences with hair professionals” [16], and “avoidance of hair professionals” [17, 18, 19, 20]. In contrast, these themes have been extensively discussed within BFRBD communities through organized self‐help groups, blog posts, private Facebook groups, and social media platforms. Additionally, two other frequently mentioned topics, drawn from anecdotal references, are “caring for the consequences of hair pulling” and “haircutting and shaving.” The first author's participation in these discussions, combined with professional experiences as a psychologist and a hair professional, has drawn attention to recurring comments and narratives from affected individuals, highlighting the importance of all five topics for them. A notable example that offers a thorough perspective is a blog post by the Canadian BFRB Self‐Help Network [21]. More recently, empirical work has begun to examine hair‐care‐related interventions, specifically a mindfulness‐based professional hair appointment for affected individuals [22].

## 3 Objectives

This exploratory study examined the intersection of HFRBDs and hair care practices by pursuing the following descriptive objectives:

Document the extent to which individuals with HFRBDs engage in constructive hair care practices and identify the nature of these practices. In doing so, the study aimed to gain a deeper understanding of how such routines may be related to symptomatic behaviors.

Describe how personal and professional hair care activities—such as washing, brushing, styling, cutting, or shaving—may influence HFRBDs. Specifically, the study investigated whether participants' experiences with these practices may temporarily interrupt pulling behaviors or help delay recurrence.

Investigate the role of hair professionals in the lives of individuals with HFRBDs, including whether salon avoidance is related to professionals' limited awareness of the condition or structural barriers such as time constraints.

## 4 Methods

The first steps in addressing the study's objectives were ensuring TTM affectedness and identifying the key hair care topics impacting those involved. To achieve this, we designed a descriptive cross‐sectional study that integrated both quantitative and qualitative evaluation methods through an online survey.

### 4.1 Procedure

Several validated instruments were selected to assess TTM affectedness. Additionally, a custom questionnaire was developed to explore participants' hair care behaviors. Then, the recruitment process was carried out. Once data collection was completed, statistical analyses were conducted.

### 4.2 Ethics Statement

The study protocol was reviewed and approved by the Ethics Committee of the Faculty of Human Sciences at the University of Cologne (Identification number: LHHF0092).

All participants provided written informed consent before participating in the study. They were informed about the purpose, procedures, risks, and benefits of the research, as well as their rights to confidentiality and the option to withdraw without penalty. The researchers ensured that all data were collected, stored, and processed in compliance with applicable data protection regulations.

## 5 Measures

Before completing the measures and a questionnaire, participants provided their demographic information, including age, gender, native language, family status, years of formal education, professional status, highest academic degree, and zip code. The subsequent measures and questionnaire were administered in the following order: The Massachusetts General Hospital Hair Pulling Scale (MGH‐HPS [23]); The Hair Care Inventory for Trichotillomania (HCI‐T); The Beliefs in Trichotillomania Scale (BiTS [24]); The Milwaukee Inventory for Subtypes of Trichotillomania‐Adult (MIST‐A [25]); The Scale for Assessing Disgust Sensitivity (SADS [26]); and the Scale for Assessing Self‐Disgust (QASD [27]).

### 5.1 The Massachusetts General Hospital Hairpulling Scale (MGH‐HPS)

The MGH‐HPS is a commonly used tool in research and therapy to assess symptoms of TTM. Developed by Keuthen et al. [23] and later adapted into German by Bohne [28], the MGH‐HPS assesses TTM symptoms through seven items: frequency of urges, intensity of urges, ability to control urges, frequency of hair pulling, attempts to resist hair pulling, control over hair pulling, and related distress. Based on the preceding 7 days, these items are rated on a five‐point Likert‐type scale (0–4), yielding a total score range of 0 to 28, where higher scores indicate more severe symptoms [28]. The scale demonstrates strong internal consistency (α = 0.89 [23]) and acceptable discriminant and convergent validity [29].

The cut‐off score for the MGH‐HPS in this study was established at 17 or higher. Individuals with a score from 0 to 16 are unlikely to receive a clinical diagnosis of TTM (e.g., refs. [30, 31]).

### 5.2 The Hair Care Inventory for Trichotillomania (HCI‐T)

The Hair Care Inventory for Trichotillomania (HCI‐T) encompasses the onset, recognition, and description of the disorder, as well as the condition and health of the hair and scalp, hair and scalp care, experiences related to touching the hair and scalp, and open‐ended questions for additional comments (see Table 1). The authors developed this questionnaire to assess the experience and significance of hair care, specifically for individuals whose HFRBDs involve their scalp hair (see Appendix).

**Table 1:** Overview of the hair care inventory for trichotillomania (HCI‐T).

Thematic Blocks	Items (Total = 73)	Subitems (Total = 57)
Onset, Recognition, and Description of the Disorder	7	
Condition and Health of Hair and Scalp	25	
Scalp		4
Hair Growth		6
Hair Texture		15
Hair and Scalp Care	33	
Washing Hair and Scalp		7
Combing and Brushing		5
Hair Styling		6
Haircutting		11
Shaving the Scalp		4
Touching Hair and Scalp	7	
Personal Additions and Comments	1	

### 5.3 The Beliefs in Trichotillomania Scale (BiTS)

The Beliefs in Trichotillomania Scale (BiTS), developed by Rehm et al. [24] and used in its German version by Hollatz and Gerlach [30], is a self‐report measure used in this study to determine if participants met the standard diagnostic criteria for TTM. In their research, Rehm et al. [24] conducted both exploratory and confirmatory factor analyses on a sample of 841 participants, which included individuals with and without self‐reported problematic hair‐pulling behaviors. Their findings supported a 14‐item scale with three distinct factors: negative self‐beliefs (NSB), low coping efficacy (LCE), and perfectionism (P). The scale demonstrated strong psychometric properties, with an overall internal consistency of Cronbach's *α* = 0.93. The three‐factor model was verified through both analyses, confirming a robust structure. Additionally, all three subscales showed significant correlations with increased hair‐pulling severity, indicating that these cognitive beliefs are closely linked to the intensity of TTM symptoms.

### 5.4 The Milwaukee Inventory for Subtypes of Trichotillomania‐Adult (MIST‐A)

The Milwaukee Inventory for Subtypes of Trichotillomania–Adult (MIST‐A), Original Version [25] is based on a comprehensive survey study (*n* = 1697) of individuals who engage in hair‐pulling behavior. In the present study, we used a German translation of the original MIST‐A. The final scale comprises 15 items that assess the TTM, particularly in focused and automatic hair‐pulling behaviors. Both the “focused” (α = 0.77) and “automatic” (α = 0.73) subscales demonstrated adequate internal consistency (Nunnally & Bernstein, 1994).

### 5.5 The Scale for Assessing Disgust Sensitivity (SADS)

The Scale for Assessing Disgust Sensitivity (SADS [26];) comprises seven items and demonstrates good internal consistency (Cronbach's α = 0.85). It exhibits moderate correlations with disgust propensity (*r* = 0.35), fear of injections (*r* = 0.20), and sociophobic tendencies (*r* = 0.28).

### 5.6 The Questionnaire for Assessing Self‐Disgust (QASD)

The Scale for Assessing Self‐Disgust (QASD [27]); comprises 20 items evaluating person‐ and behavior‐related disgust. For this study, we utilized only a nine‐item subscale focused on self‐disgust. This subscale demonstrates good internal consistency (Cronbach's α = 0.92).

Although not central to this article, measures of disgust and disgust sensitivity were included in the survey to explore potential links with HFRBDs in a broader analysis. Prior research indicates that individuals with body‐focused repetitive behaviors—particularly skin picking and trichotillomania—may have stronger disgust reactions to perceived bodily flaws, such as pimples or broken hairs. These reactions might drive the urge to remove or “correct” these features. Therefore, we aimed to collect preliminary data on disgust as a possible cognitive‐affective factor in BFRBDs. However, the analysis of these measures lies beyond the scope of the present study and will be addressed in a separate publication (compare [32, 33]).

### 5.7 Recruitment and Participation Flow

Recruitment and participation occurred from March 1, 2021, to May 1, 2023, in the German‐speaking countries of Austria, Germany, and Switzerland. The study was promoted through magazine articles, podcasts, conferences, self‐help groups, academic networks, and public and private social media platforms. Three hundred forty‐one participants accessed the survey. The initial dropout rate was 24.3%. An additional 12.9% dropped out after entering demographic data, possibly realizing they did not meet TTM criteria. Finally, 5.6% of participants, with an MGH‐HPS score of 0 indicating no current affectedness, were excluded due to concerns about data quality.

### 5.8 Sample

The final sample included *N* = 195 participants who met the inclusion criteria and completed the full survey. Participants ranged in age from 18 to 79 years (*M* = 32.4, SD = 10.8), with 93% identifying as female, 6% as male, and one individual identifying as diverse. Additional demographic and clinical characteristics are shown in Table 2.

**Table 2:** Sociodemographic characteristics of the sample (*N* = 195).

Characteristic	% (or n)
Marital Status	
Single	71%
Married	24%
Separated	2%
Divorced	3%
Widowed	1%
Native Language	
German	94%
English	3
Russian, Italian	2 each
French, Greek, Luxembourgish, Portuguese, Slovenian	1 each
Country (Zip Code)	
Germany	90%
Switzerland, Austria, Luxembourg	10%
Employment Status	
Employed	52%
Student	24%
Self‐employed	4%
Homemaker	5%
Unemployed	3%
In school	2.6%
Vocational training	2.6%
Retired	2.1%
Other (multiple)	16 responses
Disability	2.1%
Parental leave	2.1%
Officer	2.6%
Voluntary year	1.5%
Education (Years)	13.15 ± 2.1
Highest Degree (*n* = 105)	
Certificate after grade 9/10	6%
Vocational certification	2%
Higher education entrance	19%
Bachelor's degree	13%
Master's degree	15%
Doctoral degree	1%

### 5.9 Data Quality and Validity Measures

To enhance the validity of the response, the survey incorporated several quality control measures. Two clear eligibility criteria were outlined: a minimum age and relevant symptom experience. Participants had to be at least 18 years old to participate in the study, and their HFRBD had to include hair from their scalp. Clear instructions and definitions were provided to ensure participants understood key concepts. The participants received the study link via email, flyers, and social media, which was implemented on the Qualtrics survey platform. On the study's first page, participants were informed that the data collection was anonymous, that they could terminate their participation at any time without explanation, and that their data would be evaluated for research purposes. The information could also be downloaded as a PDF. Anonymity was maintained throughout to encourage honest reporting of sensitive behaviors. Open‐text questions allowed participants to provide context and enabled researchers to assess the coherence and engagement of responses. Only fully completed surveys were included in the analysis.

The time required to complete the survey likely varied, with participants spending approximately 15–20 min, depending on the length of their open‐ended responses.

### 5.10 Statistical Analyses

Data analyses were conducted using IBM SPSS Statistics Version 29.0.2.0 [34]. Participants' responses were summarized both quantitatively and qualitatively. For multiple‐choice, ranking, and yes/no questions, the data were analyzed by calculating the mean, standard deviation, and percentages of the total sample, as well as the percentage of participants who answered each specific question correctly. The qualitative analysis involved organizing responses into themes and categories.

## 6 Results

### 6.1 MGH‐HPS

Participants completed the MGH‐HPS to assess the severity of their symptoms. Scores ranged from 3 to 28, with a mean of 17.33 (SD = 5.10), a standard error of 0.36, and a median of 18. Based on the commonly used cut‐off score of 17, 118 participants (60.5%) met or exceeded the threshold, indicating probable clinical trichotillomania. The remaining 77 participants (39.5%) scored between 3 and 16, suggesting subclinical or less severe symptom levels. This distribution shows a wide range of hair‐pulling severity, indicating the inclusion of both clinical and subclinical cases in this analysis (see Table 3).

**Table 3:** MGH‐HPS cut‐off.

Participated in	*N*	MGH‐HPS 0	*N* = MGH‐HPS ≥ 17	*N* = MGH‐HPS < 17
MGH‐HPS	214	19		
HCI‐T	195		119	76

### 6.2 Hair Care Practices: Quantitative and Qualitative Results

The initial questions of the HCI‐T summarized participants' descriptions of their HFRBD, including details about their hair's density, length, and style, as well as their perceptions of their hair before and after symptom onset (see Supplement 1).

Most of the HCI‐T's questions produce findings that closely match the study's objectives. These include descriptions of the process before and after hair pulling, participants' hair care routines, and their emotional reactions to these behaviors. Additional results examine the perceived impact of hair care on pulling behavior, reasons cited for periods without pulling, and both positive and negative experiences in salon settings—particularly the reasons participants choose to avoid haircuts (see Table 4).

**Table 4:** Result summary.

Survey topics	Key findings	Objectives
The Process Before and After Hair Pulling	68.7% feel for uneven hairs; 64.6% glide fingers along hair before pulling.	How hair conditions may be related to symptomatic behaviors.
The Scalp's Condition	66.2% had or have experienced scalp issues like rashes or eczema.	How scalp conditions may be related to symptomatic behaviors.
Hair Texture	70.3% identified texture as a trigger. Many described it negatively; split hairs were common and often bothersome.	How hair conditions may be related to symptomatic behaviors.
The Participants' Hair and Scalp Care	Hair care routines varied. Many brushed/styled regularly; some used products to hide bald spots. Self‐care was preferred over care by others.	How participants engage in personal and professional hair care practices, and the nature of these routines.
Hair Care's Effect on Hair Pulling Urge	Washing and conditioning reduced urges for over half. Haircuts helped some; brushing, styling, and others' touch had little effect.	Whether hair care activities help interrupt or delay hair‐pulling behaviors.
Pull‐free Episodes After a Haircut	23.1% had pull‐free periods post‐haircut; 8.2% after shaving. Shorter hair often reduced temptation.	Whether haircuts help interrupt or delay hair‐pulling behaviors.
Positive and Negative Haircutting Experiences	Many described negative experiences with professionals due to shame or judgment. A few had positive, trusting relationships. Emotional discomfort is a key reason people avoid haircuts. Embarrassment, privacy concerns, and fear of judgment outweigh physical discomfort.	The role of hair professionals including factors contributing to salon avoidance

### 6.3 The Process Before and After Hair Pulling

Regarding the process leading to hair pulling, 33% of participants provided more detailed descriptions. The pre‐pulling phase included behaviors such as knotting, playing with, and stroking the hair (7.2%), intense looking, searching, and feeling the hair (5.6%), explicitly searching for gray hair (2.1%), and seeking out the “perfect” or “imperfect” hair (2.1%). Skin‐picking behavior on the scalp, prior to hair pulling, was reported by 1% of participants (two individuals). When asked about the tactile experience of hair pulling, 68.7% of participants said they “feel for unevenness in the individual hairs.” In comparison, 64.6% reported that they “glide along the individual hairs with their fingers.”

In open responses, 9.5% of participants mentioned trichophagia (hair eating), 7.7% specifically described nibbling, biting, chewing, and swallowing hair, and 2.1% indicated they scanned uneven hairs with their lips. Regarding pulled‐out or cut hair, one participant swallowed hair as a means of disposal, and one participant engaged in cleaning up after a hair‐pulling episode.

### 6.4 The Scalp's Condition

Half of the participants described their scalp as “flexible,” “not evoking a sensation,” “healthy,” “light,” “normal,” and “relaxed.” The other half reported sensations such as “tense,” “inflamed,” “irritated,” “warm,” “heavy,” “under pressure,” “itchy,” “crusty,” “dry,” “painful,” and “sore.” A significant portion of the sample (66.2%) reported having, or having previously experienced, 21 different scalp conditions, including rashes, eczema, and psoriasis. These conditions were linked to a higher likelihood of head‐touching behaviors. The following reasons were given by 50.3% of the participants: “My scalp itches, and I want to scratch it” (35.4%), “I feel bumps on my scalp, such as pimples or dandruff, that irritate me” (33.9%), and “My scalp is tense, and I am trying to loosen it up” (12.8%). For 27.2% of participants, their scalp condition triggered hair‐pulling behavior. Reasons included a desire to “get rid of unevenness and scabs” (11%) and the belief that “hair pulling is supposed to alleviate” the discomfort (2.6%).

### 6.5 Hair Texture

The sensation of the hair's texture was identified as a significant trigger by 70.3% of participants, with 44.1% describing it in detail using 43 different adjectives. Participants made 86 comments describing negative triggers and 63 comments describing positive ones. A total of 19% characterized the texture as “bothersome,” “broken,” “inferior,” “damaged,” “imperfect,” “shaggy,” “uneven,” “prickly,” or “different.” Additional negative triggers included sensations unrelated to the texture, such as an urge (reported by 5.1%) and a desire for symmetry and evenness (reported by 2.1%). Emotional reactions, such as “self‐hate,” “anger,” “disgust,” and “dissatisfaction,” were reported by 12.3% of participants, while physiological states, including “discomfort,” “tension,” “nervousness,” and “restlessness,” were noted by 10.3%.

Participants identified the hair shaft and root as triggers for hair pulling when perceived as “short,” “greasy,” “aching,” “thick,” or “long,” leading to an urge to remove them (6.2%). Broken or split hairs also triggered the desire to remove either the whole hair or the broken part, with 6.2% of participants reporting, “I am looking for broken hair.”

Regarding split hairs, 60% of participants noticed them, with 32.8% feeling bothered by them, 20% mildly bothered, and 7.2% not bothered. Participants reported having a few (21%), a moderate amount (20%), or a lot (19%) of split hairs. When asked about actions related to split hairs, 43.6% of participants engaged in the following behaviors: Searching for split hairs with their eyes (32.3%); pulling off the split part (31.3%); cutting off the split part (21.5%); pulling out the whole hair (19.5%); feeling for split hairs (17.9%); pulling the split hair apart (4.1%); biting off the end of the hair (2.6%); using hair oil (0.5%).

In contrast, the hair's texture and its removal evoked positive feelings. “Satisfaction,” “contentment,” “reassurance,” “relaxation,” “relief,” “well‐being,” “excitement,” “joy,” “happiness,” and “euphoria” were experienced by 12.8% of participants when they found a specific hair. The act of removal itself elicited feelings of “relief,” “satisfaction,” “triumph,” and “enjoyment” in 7.2% of the sample. Additional comments included “curiosity and interest” (3.6%) and “interesting and pleasant” (2.1%).

### 6.6 The Participants' Hair and Scalp Care

Participants reported varying hair‐washing routines: 16.4% washed their hair daily, with two participants washing it twice a day and one participant washing it three times a day. Most (73.3%) washed weekly, with 9.2% doing so 1–2 times per week, 29.2% 2–3 times, 24.6% 3–4 times, 6.2% 4–5 times, and 2.6% 5–6 times. Regarding combing or brushing, 74.9% of participants engaged in these activities, with 63.1% doing so daily and 11.8% weekly. Of those who brushed, 43.6% spent less than a minute per session, 21.5% spent about a minute, 7.2% spent 2–5 min, and 2.1% spent over 10 min. When asked why they brushed, 64.1% said to tidy their hair, 34.3% for styling, 21% to massage their scalp, and 4.1% for other reasons, such as smoothing baby hairs, covering bald spots with longer hair, removing loose hairs, preparing for braiding, or spreading sebum before washing.

Regarding styling, 36.4% styled their hair, with 26.7% using hairspray or gel, and 23.6% using a blow dryer. Among those who provided more details, 21.5% used hair clips to conceal bald spots, 5.6% used a straightening iron, and 4.1% used styling products such as mousse or paste. Most (15.4%) spent 1–5 min styling, 11.3% spent 6–10 min, and 10.3% spent 11–45 min. Two participants reported taking up to 90 min. Only 8.2% had hair treated with chemical straighteners or permanent waves before or after hair pulling started, and they said these treatments didn't affect their pulling.

Before hair pulling began, 13.8% of participants had colored or bleached their natural hair, rising to 39% afterward. Of those who colored before onset, 2.1% thought it might have contributed. Coloring or bleaching changed hair texture and reduced the urge to pull for 12.3%, worsened it slightly or significantly for 2.5%, and made no difference for 24.1%.

As for haircuts, 11.3% cut their own hair, most relied on a professional (41%), followed by a relative (5.6%), a friend (5.6%), or a partner (3.1%). Frequency varied, with 2.1% getting cuts every 4 weeks, 7.2% every 5–8 weeks, 6.2% every 9–12 weeks, 10.3% twice a year, 9.7% once a year, and 2.6% every 1.5 years or longer. Participants ranked the importance of symmetry as not at all (12.8%), a little (11.3%), medium (8.7%), or very much (15.9%).

Shaving was considered by 41.5% of the participants. A separate item indicated that 13.3% had shaved their hair. Among those who reported shaving, 3.6% did so once or twice a week, 1.5% weekly, and 2.6% monthly. One participant shaved every 2 months, another every other year.

Regarding scalp and hair touching outside of hair pulling or care, 56.4% engaged in such behaviors. These included stroking and caressing hair and scalp (39%), massaging the scalp with fingers (23.6%), placing hands on the scalp or hair (15.4%), and using a massage tool (5.6%). Additional behaviors included scratching (6.7%), skin picking (4.1%), letting hair slide through fingers without manipulating (1%), and touching or caressing a bald spot while looking for others (1%). Certain individual behaviors included aggressive stroking, twisting hair strands, padding the scalp, gently pulling hair, twisting or pulling hair across lips, and repeatedly running fingers through hair. Additionally, 33.3% reported that others touched their scalp or hair, including partners (27.2%), hair professionals (13.8%), relatives (9.7%), friends (3.6%), massage therapists (2.6%), and children (2.6%).

The next questions asked about the participants' feelings regarding their hair and scalp care (see Table 5).

**Table 5:** Participants' feelings about their hair and scalp care.

Question	Very unpleasant—unpleasant	Neutral	Pleasant—very pleasant
How does it feel when you wash your own hair?	13.8%	0.8%	45.7%
How does it feel when someone else washes your hair?	45.6%	9.2%	34.8%
How does combing/brushing feel to you?	17.5%	27.2%	30.3%
How does styling your hair feel to you?	11.3%	16.4%	8.8%
How do you experience getting your hair cut?	16.4%	11.3%	21%
How do you experience shaving your scalp?	4.1%	2.1%	7.1%
How do you experience your own touch?	8.2%	15.4%	32.8%
When someone else touches you how do you feel about the touch?	9.8%	4.6%	19%

The participants' answers regarding the influence of hair care on their hair‐pulling behavior are summarized in Table 6.

**Table 6:** Hair care's effect on hair pulling urge.

Question	Aggravates a little to a lot	Neutral	Reduces a little to a lot
Does your washing, conditioning change the urge to pull your hair?	2.6%	41.5%	54.1%
Does your combing/brushing change the urge to pull your hair?	7.7%	51.8%	15.4%
Does your styling change the urge to pull your hair?	7.9%	20%	8.7%
Does haircutting change the urge to pull your hair?	4.1%	19%	34.1%
Does shaving change the urge to pull your hair?	0%	3.1%	10.3%
Does the touch of another person (stroking, combing, brushing, washing, massaging) change the urge to pull your hair?	3.1%	20.5%	9.8%
Does your own touch not related to hair care or hair pulling change the urge to pull your hair?	27.7%	19%	9.7%

### 6.7 Pull‐Free Episodes After a Haircut

A pull‐free episode following a haircut was reported by 23.1% of participants. The duration of these episodes varied: 5.1% experienced pull‐free periods lasting 20 min to 1 week, 9.2% reported 2–12 weeks, and 4.1% noted durations ranging from 11 weeks to several years. Responses to multiple‐choice questions about the reasons behind these episodes highlighted feelings of improved scalp and hair health, a sense of orderliness, and a lighter sensation. One participant wrote: “After haircutting, I always manage not to pull for a while.”

A pull‐free episode following scalp shaving was reported by 8.2% of participants. This episode lasted 10 years for two participants, although they noted it involved other contributing factors. For 3.1% of participants, the pull‐free period ranged from 1.5 months to several months. Three participants explained that the urge to pull subsided until their hair reached a certain length again, with one stating, “As long as the hairs are too short to be wrapped around my finger,” and one participant reported, “I do not pull when my hair is shaved.”

Additionally, participants cited emotional factors, such as feeling more attractive, self‐confident, and beautiful, as well as feeling free after a haircut. For 13.3% of participants, a desire not to disrupt the newly styled appearance was also a motivating factor (see Table 7).

**Table 7:** Participants' reasons for pull‐free episodes after a haircut.

How do you explain the pull‐free episode?	not at all to a little	medium	Quite a bit to very much
“When the hair is shorter, I pull my hair less.”	10.8%	1%	10.3%
“My scalp and hair felt:			
orderly	8.2%	2.6%	11.3%
light	7.2%	3.1%	10.8%
perfect	9.8%	3.6%	8.2%
free	10.7%	3.1%	7.7%
healthy.”	6.2%	3.1%	12.8%
“All in all, I felt:			
attractive	6.2%	3.6%	12.3%
beautiful	6.2%	4.1%	11.8%
self‐confident	6.2%	3.6%	11.3%
freed.”	6.7%	4.6%	10.3%
“I didn't want to mess up or destroy the newly created work.”	4.6%	3.6%	13.3%

Additional reasons included experiences such as: “I was treated kindly despite my bald spots. I felt relieved, and the tension and shame began to subside. With the support of a group and encouragement from a partner, I kept an Excel sheet to track my hair‐pulling behavior. Due to its length, I could no longer see the hair, which motivated me to make a fresh start and stop pulling. Split ends were no longer noticeable or visible.”

The survey's final open‐ended question invited participants to share anything related to their hair or HFRBDs. Ten responses about helpful strategies focused on the length, health, styling, and care of their hair. Specific strategies included shaving, keeping hair short, wearing it in a ponytail, using a tight headscarf, incorporating partial dreadlocks, using hairpieces, and maintaining well‐cared‐for, freshly styled, washed, cut, and healthy hair. Participants also mentioned avoiding certain hair products while preferring others.

Out of the sample, 4.6% of participants reported pulling out their hair more after a haircut. Three participants cited the newly created work as intolerable. This expresses a strong adverse reaction to the new haircut, clearly depicting that changes in hair's look or feel can provoke sensory or emotional discomfort rather than satisfaction. One experienced increased stress, and two explained, “I looked at my hair in the mirror for a long time and noticed more hair I felt compelled to pull out.” One participant mentioned, “The hairspray and gel made my hair feel different, which made me want to pull it out.”

### 6.8 Positive and Negative Haircutting Experiences

The open‐ended question about haircutting experiences received responses from 35.4% of the sample. The answers explained why participants were unwilling to let someone else (17.9%) or a hair professional (27.2%) cut their hair, as shown in Table 8.

**Table 8:** Reasons for not receiving a haircut.

Why do you not let another person cut your hair?	not at all to a little	medium	Quite a bit to very much
“I am embarrassed to show other people my scalp and hair.”	6.1%	3.1%	25.1%
“I don't want to have to explain my bald spots.”	9.8%	1%	23.6%
“I don't want to lie.”	12.3%	8.2%	16.4%
“When others touch my scalp and hair, it feels painful.”	29.7%	5.1%	6%
Why do you not let a hair professional cut your hair?			
“I find it difficult to show my scalp and hair to a stranger.”	18.5%	6.2%	43.5%
“I don't like to see myself in the mirror.”	48.2%	7.2%	12.4%
“There is no privacy. Other people could see my scalp and hair.”	33.3%	5.6%	28.7%
“The salon atmosphere is too hectic and loud for me.”	47.7%	5.1%	14.8%

Negative salon experiences, represented by 54 statements, were primarily related to the interaction between the hair professional and the individual. Participants described communication during past haircuts as containing unpleasant questions, comments, judgments, unsolicited advice, and blame, mainly due to the hair professional's lack of understanding of the disorder. As a result, they felt observed, exposed, uncomfortable, embarrassed, ashamed, humiliated, fearful, resigned, guilty, and frustrated. They explained that they refrained from letting anyone cut their hair because they would have to explain the disorder, wanted to avoid unwanted questions and judgments, or could not bring themselves to share the truth. Additionally, six participants reported that their hair was cut too short.

Positive salon experiences were reflected in four statements describing satisfying results, a common occurrence among salon‐goers. One participant shared, “The outcome of the hairstyle was perfect.” Additionally, two participants described finding a hair professional with whom they felt comfortable despite their hair‐pulling behavior. One participant noted, “We can talk about my disorder openly now, and she has found a sensitive way to handle it.”

## 7 Discussion

This study aimed to investigate constructive hair care practices among individuals with hair‐focused repetitive behavior disorders (HFRBDs) and to explore the relationship between these routines and their symptoms. The results confirmed that participants do engage in a wide range of hair care behaviors, including washing, brushing, cutting, and styling. Many of these behaviors are experienced as pleasant or neutral, and some are associated with a reduction in hair‐pulling urges. Hair texture, scalp sensations, and the desire for symmetry emerged as common triggers. Emotional and physical responses to hair and scalp condition reportedly played a relevant role in hair‐pulling behavior. Finally, the study examined the role of hair professionals in the participants' lives and found mixed experiences, emphasizing the need for individualized, respectful engagement.

### 7.1 Hair Appearance as a Clinical Consideration

Participants confirmed an expected noticeable decline in hair quality and density after onset of TTM. However, their detailed descriptions of hair indicate that the disorder does not have a clear or consistent visual appearance. Its effects can be seen through bald patches and uneven hair lengths, or hidden beneath a thick layer of hair. Considering whether the effects are visible or concealed may be important when developing hair care strategies to meet each individual's unique needs.

### 7.2 Constructive Hair Care and Sensory Triggers

The participants described their scalp and hair conditions and outlined the process of pulling hair. Key factors for pulling include tactile triggers such as hair texture, scalp sensations, and the desire for symmetry.

The participants' detailed descriptions of the process leading up to hair pulling suggest that hair texture and scalp condition are central triggers. Hair texture was identified as a trigger by 58.5% of participants, and for 89.7%, this trigger evoked either a positive or negative emotional response. Split ends were noticed by 60%, with 32.8% reporting that they were bothered by them. Affected individuals often scan for unhealthy or unusual hairs, categorize them as “bad,” and experience a sense of satisfaction when they find and ultimately remove them. These findings suggest that more than half of the participants may be susceptible to hair texture, which could inform future research aimed at subgrouping individuals based on their specific triggers.

### 7.3 Scalp Condition, Discomfort, and Urge to Pull

A significant proportion of participants reported that their urge to touch or pull their hair was associated with scalp discomfort, including itchiness and pain. Half reported scalp irritation as a touch trigger, and over a quarter identified it as a direct pulling trigger. Although no baseline comparison was available, these results suggest scalp health may play an underrecognized role in the symptom cycle of HFRBDs. To the best of our knowledge, prior literature has not addressed scalp condition in BFRBD research; however, these findings suggest that dermatological assessment and scalp‐specific care may be a meaningful adjunct to psychological interventions. This raises the broader clinical question: Could improving scalp and hair health reduce hair‐pulling behavior? While causal relationships remain speculative, these findings warrant further study into integrated models of care that include dermatology and behavioral health.

Participants' narratives about touching their scalps—describing sensations ranging from soothing to irritating—highlight the importance of tactile experience in HFRBDs. These accounts align with the Sensory (S) domain of the comprehensive behavioral model [35], which conceptualizes BFRBDs as being maintained in part by sensory reinforcement. For some individuals, the texture of broken hairs, dryness, or scalp tension appeared to elicit discomfort and urge‐driven behaviors. In contrast, others reported a calming or grounding effect from scalp contact or brushing their hair. This underscores the potential clinical utility of targeting sensory triggers and sensory‐based regulation strategies in treatment planning.

### 7.4 Sensory Gratification, Behavioral Substitution, and Intervention

Another noteworthy observation is that the study yielded 49 comments expressing positive feelings when a specific hair was found and 14 comments indicating that removing hair evoked positive feelings. Arguably, these factors indicate reward and may partially reinforce the repetitive behavior. Practically, if an individual experiences joy from scanning for particular textures, they might benefit from using a textured fidget toy. Conversely, for those who derive satisfaction from pulling, a pull‐it or pop‐it toy could serve as a healthier alternative to satisfy that urge.

Viewing HFRBDs as a potentially lengthy, ritualistic process, as described by this study's participants—rather than a singular impulsive act—opens up more possibilities for targeted interventions. For instance, an individual might search for and find a similar textural sensation on their body or use a fidget toy as a substitute. In the context of Habit‐Reversal Therapy, the individual could interrupt the hair‐pulling sequence not when their hand reaches their head but after the scanning phase, before the pulling occurs. This may be a more manageable step, as the person only needs to alter the latter, more destructive part of the sequence rather than abandoning the entire behavior.

A practical intervention could involve providing personal hair care education to help individuals better understand the hair growth process, its natural characteristics, and common medical conditions affecting hair.

### 7.5 Unanticipated Findings and Diagnostic Confusion

An unexpectedly high proportion of participants (9.7%) reported having alopecia areata. Although the general population prevalence of alopecia areata is often cited as approximately 2%, this figure can vary depending on age, gender, or reporting method and does not specifically apply to this sample (see ref. [36] for general prevalence estimates). Several explanations are possible. One is that some individuals might confuse hair loss due to trichotillomania with alopecia areata, especially if they lack a formal diagnosis or adequate public awareness of BFRBDs. Alternatively, individuals with hair loss may be more interested in research related to hair loss or hair‐pulling behaviors, which could lead to a higher representation in this sample. We emphasize that no definitive conclusions about comorbidity or diagnostic overlap can be drawn from these data; however, the findings underscore the need for enhanced public understanding and improved differential diagnosis of hair loss conditions.

Additionally, regarding the tendency to judge specific hairs as “negative,” individuals could benefit from education about how the cosmetic industry reinforces these judgments. The industry creates unrealistic standards by promoting the ideal of “perfect” hair, often achieved through digitally enhanced images, extensions, and chemical treatments. With comprehensive health education and personal awareness, affected individuals could feel more empowered to care for their hair constructively, ultimately reducing or eliminating negative judgments about it.

More than half of the participants (56.4%) engage in additional hair‐related behaviors—such as caressing and massaging their hair—alongside their hair care and hair‐pulling habits, viewing these actions as a form of self‐care. Furthermore, 32.8% reported experiencing self‐touch as pleasant to very pleasant. This suggests that affected individuals may have positive associations with their hair, which could be crucial for their willingness to address their condition and take the next steps in their recovery. However, there is a risk that regular self‐soothing behaviors, due to their proximity and frequency, may inadvertently trigger the hair‐pulling behavior.

Our second objective was to explore whether aspects of individuals' hair care routines influenced their urge to pull. Participants reported that washing, brushing, cutting, and shaving had a slight to moderate reduction in hair‐pulling behaviors, while perming, coloring, and styling had no apparent effect.

While washing and brushing one's hair are straightforward interventions, it should not be assumed that affected individuals have necessarily considered them as such. Consequently, our findings recommend incorporating these hair care practices into a broader treatment approach. Emotional factors—such as feeling more attractive and self‐confident—are more commonly linked to pull‐free episodes than physical sensations like having a healthier or more orderly scalp and hair, with 23.1% of participants reporting this association. These results suggest that emotional and physical factors warrant further investigation to understand their impact on symptom reduction better. HFRBDs involving head, beard, and body hair are unique among BFRBDs because they allow for the voluntary removal of the affected area without causing physical harm. Shaving the source of distress is a logical approach. Nearly half of the participants, 41.5%, considered shaving, and 13.3% had shaved their hair at least once, with 8.2% reporting a period of no hair‐pulling afterward. However, these numbers indicate that shaving may only be a solution for a small subset of individuals. This idea is reinforced by 73.4% of the study's participants, who described their hair as medium‐long to very long, reflecting their preferences in hair length. In hindsight, it would have been helpful to include additional questions exploring the reasons behind participants' choices of hair length and why shaving might not be a viable option for them.

### 7.6 Involvement of Hair Professionals

Our third objective was to explore whether including hair care professionals in recovery might be beneficial. Participants specified that having their hair washed by another person was unpleasant to very unpleasant. These findings could prove helpful, as the individual's support team, particularly hair professionals, could ask the affected individual about their preferences and, where possible, offer to cut their hair without washing it to enhance comfort and reduce distress.

Interestingly, despite negative experiences at salons reported in previous research [21] and echoed in this sample, nearly half of the participants allowed someone other than themselves to cut their hair. The presence of a mirror and the potentially loud, hectic salon environment did not deter participants from visiting. Responding to the open‐ended questions, none of the participants preferred more privacy or additional time during salon visits. However, the Likert scale responses revealed a more nuanced picture: a third of the participants felt that privacy was not a significant issue, and another third of the sample indicated that a lack of privacy would be a barrier to visiting a hair professional. With appropriate encouragement and communication strategies, requesting and arranging privacy in a professional salon setting for those who prefer it could be easily implemented. This consideration could be an essential aspect of counseling affected individuals, helping them feel more comfortable during salon visits.

### 7.7 Behavioral Reinforcement and Intervention Opportunities

The findings can be viewed in terms of whether constructive hair care activities (e.g., engaging in mindful haircut appointments) might serve as *alternative reinforcers* rather than *complementary reinforcers* to hair pulling. Alternative reinforcers provide a competing source of reward that may reduce engagement in the problematic behavior, whereas complementary reinforcers could be associated with or even trigger that behavior. Here, participants reported positive emotional responses following a haircut appointment, which suggests the potential of hair care interventions to support self‐regulation and reduce hair‐pulling urges through increased reward sensitivity to constructive routines. While causality cannot be inferred, these patterns may inform future intervention models that integrate pleasurable body care experiences as part of harm reduction or behavior substitution strategies. These interpretations are consistent with recent findings showing that structured mindfulness‐based professional hair appointments were accompanied by reductions in hair‐pulling urges and time spent pulling hair [35]. Together these findings suggest that constructive hair care experiences may function as alternative reinforcers when delivered in structured, supportive manner by informed hair professionals.

### 7.8 Limitations

This study has several limitations. First, it faces common issues with internet‐based samples, such as self‐selection bias and limited applicability to individuals who may not participate in online communities or who are not interested in hair care.

The study's participants might differ from the broader population of individuals with HFRBDs, possibly representing a distinct subgroup with specific coping strategies or openness to treatment.

The main limitation of this study is the lack of a formal clinical diagnosis of TTM and related behaviors, such as through a structured diagnostic interview; instead, symptom severity scores from the MGH‐HPS were used to help interpret clinical features. Because the goal was to understand how individuals who self‐identify as hair pullers experience professional hair care, we did not specifically differentiate between the two groups, defined by the MGH‐HPS cut‐off. Future research should differentiate between participants with a clinical diagnosis and also evaluate relevant comorbidities to improve the robustness and clinical validity of the findings.

Third, the absence of a control group prevents a comparative analysis of hair care habits or preferences. While some behaviors reported (e.g., trichophagia) are highly specific to the clinical group, having comparative data would have helped put other findings, such as hairstyle preferences or hair care habits, into context. Without reference data from the general population, it is hard to determine whether specific patterns (e.g., frequency of hair washing or wearing hair in a ponytail) are unique to individuals with hair‐pulling behaviors or simply reflect broader trends.

It is also essential to note that participants' reflections on how specific hair care practices influence their hair‐pulling urges were based on retrospective self‐report, without the benefit of real‐time tracking or ecological momentary assessment (EMA). As such, the accuracy of these perceived associations may be limited by recall bias or post hoc rationalization. Nevertheless, these self‐reported patterns offer valuable preliminary insights. They may serve as hypotheses for future clinical research employing contemporaneous data collection methods to more rigorously test the impact of hair care behaviors on symptom fluctuations.

Similarly, some participants reflected on how their perception of their hair changed following the onset of hair‐pulling behaviors. These retrospective accounts are constrained by limited temporal precision, particularly given the frequent early onset and the absence of clear before/after markers in much of the sample. The samples' ages ranged from 18 to 79 years, with a mean of 32.4 years. Since 87.7% of participants reported an identifiable age or time of onset between 2 and 39 years, with a mean of 13.5 years, most of the onsets occurred during childhood. This clearly limits the reliability of retrospective comparisons, particularly given potential recall bias and the interpretive challenges associated with reconstructing perceptions from a very young age. For this reason, we decided not to conduct statistical analyses on these descriptors. Future research, using longitudinal or real‐time methods, may help clarify how hair perception evolves during the development and maintenance of the disorder.

Also, we did not systematically explore the impact of hair replacements such as wigs or extensions. Three participants mentioned hair replacements, which represent distinct, specialized solutions that were outside the scope of the current investigation.

Lastly, while the current analysis focused on descriptive patterns within domains, future research may benefit from examining potential associations between participants' self‐perceptions of hair characteristics and their affective responses to hair/scalp care (Table 5), as well as the impact of care routines on pulling urges (Table 6). Such analyses could help clarify whether particular cognitive or emotional profiles are linked to specific behavioral outcomes.

### 7.9 Future Directions

To build on these findings, future research should utilize larger, diagnostically confirmed samples, comparison groups, and real‐time data collection methods, such as ecological momentary assessment (EMA). Examining how self‐perceptions of hair and scalp evolve, primarily through longitudinal studies, would also deepen our understanding. Additionally, developing and testing comprehensive interventions that combine behavioral therapy, sensory modulation, and dermatological care could provide more holistic pathways to recovery.

## 8 Conclusion

The health of their scalp and hair can be essential for some individuals with HFRBDs. While the assumption that healthier, well‐cared‐for hair might reduce pulling behaviors cannot be conclusively proven, incorporating personal and professional hair care into therapeutic interventions may benefit some individuals.

A significant barrier for individuals with HFRBDs seeking salon services is the lack of knowledge among hair professionals about the disorder. This knowledge gap often causes affected individuals to experience shame, feel judged, and be misunderstood. Hair professionals must be informed about the disorder to foster a more inclusive environment. Integrating education about all BFRBDs into vocational training programs would be a pivotal first step in addressing this need. By raising awareness and promoting empathy, hair professionals can play a vital role in normalizing the experiences of those with HFRBDs and ensuring they feel respected and understood.

## Author Contributions

**Linda Hollatz:** conceptualization, data curation, formal analysis, methodology, project administration, writing – original draft, writing – review and editing. **Alexander L. Gerlach:** conceptualization, data curation, formal analysis, methodology, software, supervision, validation, writing – review and editing.

## Ethics Statement

The study protocol was reviewed and approved by the Ethics Committee of the Faculty of Human Sciences at the University of Cologne (Identification number: LHHF0092). All participants provided written informed consent prior to their participation in the study. They were informed about the purpose, procedures, risks, and benefits of the research, as well as their rights to confidentiality and the option to withdraw without penalty. The researchers ensured that all data were collected, stored, and processed in compliance with applicable data protection regulations.

## Conflicts of Interest

The authors declare no conflicts of interest.

## Transparency Statement

The lead author Linda Hollatz affirms that this manuscript is an honest, accurate, and transparent account of the study being reported; that no important aspects of the study have been omitted; and that any discrepancies from the study as planned (and, if relevant, registered) have been explained.

## Supporting information


**Table S1:** Summary of the Initial Results. **Table S2:** Participant Describing Their Hair Before and After the Onset.

## Data Availability

The data that support the findings of this study are available on request from the corresponding author. The data are not publicly available due to privacy or ethical restrictions.

## Appendix

**HCI‐T English—Hair Care Inventory for Trichotillomania**

Questionnaire regarding Personal and Professional Hair Care for Individuals with Hair‐Focused Repetitive Behaviors.

**Onset, Recognition, Course, and Specificity of the Disease**

1. When did you start pulling your head hair?
2. What hair do you pull out other than the hair on your head?
  No other hair.
  Eyebrows.
  Eyelashes.
  Hair of the Beard.
  Armpit hair,
  Body hair on arms, legs, chest.
  Pubic hair.
  Other, namely.
3. Describe the process before and after you pull a hair out.
  1. I slide along the individual hair with my fingers.
  2. I feel for unevenness in the individual hair.
  3. Other, namely.
4. Do you try to establish symmetry when pulling your hair?
  **Table:**
  0	1	2	3
  not at all	a little bit	medium	very
5. Do you engage into other body‐focused repetitive behaviors? yes/no.
  Which other body‐focused repetitive behaviors do you engage in?
    1. Nail biting.
    2. Skin picking.
    3. Cheek biting.
    4. Other, namely.
  How often do you find yourself engaging in these behaviors?
  Never/Daily _x/Weekly _x/Monthly _x.
6. When did you realize that hair pulling has become problematic?
7. Is there anything else you would like to tell us about your relationship with your hair and your condition?
  **Condition and Health of Hair and Scalp**
  **Scalp**
8. Please check the items that you have already experienced or are currently experiencing:
  1. Diffuse hair loss/Alopecia areata/androgenic alopecia
  2. Scalp rashes with/without itching
  3. Psoriasis with dry/greasy scales
  4. Neurodermatitis: eczema with/without itching
  5. Warts on the scalp
  6. Scars on the scalp
  7. Other, namely
9. How does your scalp feel?
  No sensation/tense/inflamed/relaxed/flexible/light/heavy
  Other, namely
10. Is there any scalp condition that makes you more likely to touch your head? yes/no
  If yes…then because
  1. my scalp itches and I want to scratch myself.
  2. my scalp is tense and I am trying to loosen it.
  3. I feel bumps on my scalp, e.g. pimples, dandruff that irritate me.
  4. Other condition:
11. Does the condition of your scalp trigger hair pulling? yes/no
  If yes, can you describe it in more detail?
  **Hair Growth**
12. How would you describe your natural hair density (f.e. sparse, normal, abundant, growing strongly)?
13. How long is the majority of your head hair?
  1. Very short (almost shaved, shorter than 3 cm/1 inch)
  2. Short (above the ears, above the chin)
  3. Medium length (between chin and shoulders)
  4. Long (reaching over the shoulders)
  5. In your words:
14. Do you currently have areas on your scalp where hair grows back and is visibly shorter than the surrounding hair? yes/no
15. Do you currently have areas on your scalp where no hair can be seen? yes/no
16. Instead of bald spots, do you tend to have less hair overall due to hair loss/hair pulling? yes/no
  If yes…
  Do you have less hair overall because…
  hair loss
  hair pulling
  Other reason
17. Do you currently have areas on your scalp where hair no longer grows? yes/no
  If yes, do you know the reason?
  Scarred skin
  Alopecia areata
  Other reason
  **Hair Texture**
  The following is about the texture (Latin textura—tissue) of your hair in general and the hair, which you may preferably pull out: the surface of the hair, the hair as a whole and its shape. To distinguish between the terms texture and structure: The structure of a hair refers to its construction: the root and the hair shaft. (Latin: structura—building).
18. How would you describe your head hair before the onset of trichotillomania:
  fine/thin/sparse/robust/strong/dry/split/oily/silky/shiny/scruffy/frizzy/___
19. How would you describe your head hair now:
  fine/thin/sparse/robust/strong/dry/split/oily/silky/shiny/scruffy/frizzy/___
20. How is your hair naturally shaped?
  smooth/wavy/curly/extremely curly/___?
21. Did the shape of your hair change during the course of your life by itself? yes/no
  If yes then how?
  from smooth to wavy
  from smooth to curly
  from smooth to extremely curly
  from wavy to curly
  from wavy to extremely curly
  from curly to extremely curly
  from extremely curly to curly
  from extremely curly to wavy
  from extremely curly to smooth
  from curly to wavy
  from curly to smooth
  from wavy to smooth
  In your words:
22. Did the texture of your hair change during trichotillomania by itself? yes/no
  If yes then how?
  from smooth to wavy
  from smooth to curly
  from smooth to extremely curly
  from wavy to curly
  from wavy to extremely curly
  from curly to extremely curly
  from extremely curly to curly
  from extremely curly to wavy
  from extremely curly to smooth
  from curly to wavy
  from curly to smooth
  from wavy to smooth
  In your words:
23. Is the texture of your hair a trigger for pulling out your hair? yes/no
24. Does the hair you pull out have a specific texture? yes/no
  If yes, please describe the texture of the hair, which contributes to you pulling it out:
25. Does the texture trigger a sensation? yes/no
  Describe the sensation triggered by the texture:
  In your words:
26. Do you find specific hair disgusting? yes/no
  Which kind of hair do you find disgusting?
  How disgusting do you find the hair?
  **Table:**
  1	2	3
  a little bit	medium	very
27. Do you find it disgusting, when you pull your hair out? yes/no
  How disgusting do you find it, when you pull your hair out?
  **Table:**
  1	2	3
  a little bit	medium	very
28. Did you change your hair with perms or chemical straightening before and/or after the onset of trichotillomania? yes/no
  If yes then how?
  from smooth to wavy
  from smooth to curly
  from smooth to extremely curly
  from wavy to curly
  from wavy to extremely curly
  from curly to extremely curly
  from extremely curly to curly
  from extremely curly to wavy
  from extremely curly to smooth
  from curly to wavy
  from curly to smooth
  from wavy to smooth
  In your words:
  If before…
  Did the texture of the hair changed by the chemical treatment start the onset of trichotillomania? yes/maybe it contributed to it/no
29. Does the texture of the hair changed by the chemical treatment affect your hair pulling urge?
  **Table:**
  −2	−1	0	1	2
  aggravates a lot	aggravates a little	neutral	reduces a little	Reduces a lot
30. Have you changed your natural hair color before or/and after the onset of trichotillomania by bleaching/coloring? yes/no
  If before…
  Did the texture of the hair changed by the chemical treatment start the onset of trichotillomania? yes/maybe it contributed to it/no
31. Does the texture of the hair that has been changed by dyeing have an impact on your hair pulling urge?
  **Table:**
  −2	−1	0	1	2
  aggravates a lot	aggravates a little	neutral	reduces a little	Reduces a lot
32. Have you noticed split hair? yes/no
  If yes, how many split hairs do you have?
  few/some/a lot?
  If you have split hair, does the split hair bother you? yes/a little bit/no?
  If you notice split hair, do you do something with the split hair? yes/no
  If yes, what do you do exactly?
  1. I search for split hair.
  2. I feel for split hair.
  3. I pull out the whole hair, which is split.
  4. I pull off the split part.
  5. I cut the split part off.
  6. In your words:
  **Hair and Scalp Care**
  **Washing Hair and Scalp**
33. How often do you wash your scalp and hair?
  Never/Daily _x/Weekly _x/Monthly _x
34. With what do you wash your scalp and hair?
  Only with water/with a shampoo/with a hair soap/___
35. Do you use conditioner? yes/no
  If yes, then what?
  Creme rinse/herbal tea rinse/apple cider vinegar/___
36. Do you sometimes us hair repair treatments or apply oil treatments? yes/no
  If yes what exactly do you use?
37. How does it feel, when you wash/condition/care for your scalp and hair?
  **Table:**
  −2	−1	0	1	2
  Very unpleasant	unpleasant	neutral	pleasant	very pleasant
38. How does it feel, when you get your scalp and hair washed/conditioned/cared for by another person?
  **Table:**
  −2	−1	0	1	2
  Very unpleasant	unpleasant	neutral	pleasant	very pleasant
39. Does the washing/conditioning/other treatment of your scalp and hair change the urge to pull your hair?
  **Table:**
  −2	−1	0	1	2
  aggravates a lot	aggravates a little	neutral	reduces a little	Reduces a lot
  **Combing and Brushing**
40. Do you comb or brush your hair? yes/no
  Daily _x/Weekly _x/Monthly _x
41. When you comb or brush your hair, do you do it to bring order into the hair/to style it/to massage the scalp/____?
42. How long do you comb/brush per occasion?
  Less than a minute/about a minute/two to 5 min/___
43. How does the combing/brushing feel to you?
  **Table:**
  −2	−1	0	1	2
  Very unpleasant	unpleasant	neutral	pleasant	very pleasant
44. Does the combing/brushing change the urge to pull your hair?
  **Table:**
  −2	−1	0	1	2
  aggravates a lot	aggravates a little	neutral	reduces a little	Reduces a lot
  **Hair Styling**
45. How do you like to wear your hair the most?
  Loose/covered/tied together in a bun or pony‐tail/pinned up/braided/
  In your words:
46. Do you use hair pieces/a wig? yes/no
47. Do you style your hair? yes/no
  1. If yes, do you use hair gel/hairspray to bring and hold your hair into a specific shape? yes/no
  2. Do you blow‐dry your hair to bring and hold your hair into a specific shape? yes/no?
  3. How do you style your hair:
48. How much time do you spend daily with styling your hair? x minutes
49. How does the styling feel to you?
  **Table:**
  −2	−1	0	1	2
  Very unpleasant	unpleasant	neutral	pleasant	very pleasant
50. Does the styling of your hair change the urge to pull your hair?
  **Table:**
  −2	−1	0	1	2
  aggravates a lot	aggravates a little	neutral	reduces a little	Reduces a lot
  **Haircutting**
51. 51. Did you have special haircutting experiences that mean something to you in relation to your hair? yes/no If yes, please explain:
52. Do you let someone cut your hair since the onset of trichotillomania? yes/no
53. If yes, who usually cuts your hair?
  You yourself, hairdresser, partner, relative, friend,____
  How often is your hair being cut? every x days
54. Do you aim for symmetry when cutting your hair?
  **Table:**
  0	1	2	3
  not at all	a little bit	medium	very
55. How do you experience the cutting?
  **Table:**
  −2	−1	0	1	2
  Very unpleasant	unpleasant	neutral	pleasant	very pleasant
56. If you don't let your hair cut by another person, what are the reasons?
  1. I am embarrassed to show other people my scalp and hair.
  2. I don't want to have to explain my bald spots.
  3. I don't want to lie.
  4. When others touch my scalp and hair, it feels painful.
  5. Other reasons/in your words:
57. Based on your answers to the previous questions, whether you have your hair cut at all and if so by whom, it can be deducted that you will not let a hairdresser cut your hair. Please explain this
  1. I find it difficult to show my scalp and hair to a stranger.
  2. I would go to a hairdresser, but I don't like to see myself in the mirror.
  3. I would go to a hairdresser, but there is no privacy and other people could see my scalp and hair.
  4. I would go to a hairdresser, but the salon atmosphere is too hectic and loud for me.
  5. Other reasons/in your words:
58. Does the haircutting change the urge to pull your hair?
  **Table:**
  −2	−1	0	1	2
  aggravates a lot	aggravates a little	neutral	reduces a little	Reduces a lot
59. Have you ever had a pull‐free episode after a haircut? yes/no If yes, how long did this period last? x days
60. If you had a pull‐free episode after a haircut, how do you explain it?
  1. When the hair is shorter, I pull my hair less.
  2. My scalp and hair felt orderly.
  3. My scalp and hair felt light.
  4. My scalp and hair felt perfect.
  5. My scalp and hair felt free.
  6. My scalp and hair felt healthy.
  7. All in all, I felt attractive.
  8. All in all, I felt beautiful.
  9. All in all, I felt self‐confident.
  10. All in all, I felt freed.
  11. I didn't want to mess up or destroy the newly created work.
  12. Other reasons, in your words:
61. Have you ever after a haircut pulled out your hair more? yes/no
  How long did this phase last in days?
  How do you explain it?
  1. On my head I felt:
  2. All in all, I felt:
  3. I couldn't stand the newly created work.
  4. Other reasons, in your words:
  **Head Shave**
62. Do you shave your head since the onset of trichotillomania? yes/no
  If yes how often? every x days
  If never, have you ever thought of shaving off all the hair on your head? yes/no
63. How do you experience the shaving?
  **Table:**
  −2	−1	0	1	2
  Very unpleasant	unpleasant	neutral	pleasant	very pleasant
64. Does shaving change the urge to pull your hair?
  **Table:**
  −2	−1	0	1	2
  aggravates a lot	aggravates a little	neutral	reduces a little	Reduces a lot
65. Have you experienced a pull‐free period after shaving?
  How long did it last in days?
  **Touching the Scalp and Hair**
66. Do you touch your scalp and hair in a way that is not related to hair care or hair pulling? yes/no. What exactly do you do?
  1. I stroke and caress my hair and scalp with my hands.
  2. I massage my scalp with my fingers/a massage tool.
  3. I lay my hands on my scalp and hair.
  4. Other in your words:
67. How do you experience your own touch?
  **Table:**
  −2	−1	0	1	2
  Very unpleasant	unpleasant	neutral	pleasant	very pleasant
68. Does your own touch not related to hair care or hair pulling change the urge to pull your hair?
  **Table:**
  −2	−1	0	1	2
  aggravates a lot	aggravates a little	neutral	reduces a little	Reduces a lot
69. Does someone else but you touch your head and scalp hair? yes/no
70. Who but you touches your head and your scalp hair?
  Partner/relative/friend/hairdresser/massage‐therapist/___
71. When someone else touches you, how do you feel about the touch, stroking/combing—brushing/washing/massaging?
  **Table:**
  −2	−1	0	1	2
  Very unpleasant	unpleasant	neutral	pleasant	very pleasant
72. Does the touch, stroking/combing—brushing/washing/massaging of another person change the urge to pull your hair?
  **Table:**
  −2	−1	0	1	2
  aggravates a lot	aggravates a little	neutral	reduces a little	Reduces a lot
  **Personal Additions and Comments**
73. Is there anything else you would like to share with us about your relationship with your hair and your hair care?

**Thank you!**
