# Supplementary material for: Hair Care and Hair‐Focused Repetitive Behaviors: A Descriptive Cross‐Sectional Study
Source: Health Sci Rep. 2026 Jan 20;9(1):e71730. doi: 10.1002/hsr2.71730 (PMC12819159; doi:10.1002/hsr2.71730)
Supplement: Supplementary file 1 — Table S1: Summary of the Initial Results. Table S2: Participant Describing Their Hair Before and After the Onset. [file HSR2-9-e71730-s001.docx]

**Supplement 1**

**Self-Reported Experiences of HFRBDs**

The initial questions of the HCI-T summarized participants’ descriptions of their HFRBD, including details about their hair's density, length, and style, as well as their perceptions of their hair before and after symptom onset **(see Table S1).**

**Table S1**

*Summary of the Initial Results*

| Survey Topics | Findings |
| --- | --- |
| Self-Reported Experiences of HFRBDs | Hair-pulling began between ages 2–39 (avg. 13.15); became problematic around 17.6. Nearly half linked it to symmetry; 28.7% reported non-TTM HFRBDs. |
| Participants’ Scalp and Hair Characteristics | Over half described their hair as abundant; many had long hair.  Most reported regrowth, thinning, or bald patches due to pulling. |
| Participant Describing Their Hair Before and After the Onset | Participants noticed a decline in hair quality and density after onset. |

The average age at which participants first began hair-pulling ranged from 2 to 39 years, with a mean of 13.15 years (*SD* = 6.5). Participants reported that the behavior became problematic, on average, at 17.6 years, often coinciding with the appearance of visible bald spots, comments from others, or increased intensity of the behavior. All participants engaged in scalp hair pulling, with 33.3% also pulling pubic hair, 26.7% pulling eyebrow hair, 23.6% pulling eyelashes, 14.9% pulling body hair, 6.7% pulling armpit hair, and 5.6% pulling beard hair.

Regarding symmetry, 53.7% of participants did not attempt to create symmetry when pulling hair, while 46.3% associated hair-pulling with a desire for symmetry: 24.5% slightly, 12.2% moderately, and 9.6% to a great extent.

Additionally, 21.5% of participants reported cutting their split ends, which could suggest Trichotemnomania (hair cutting). One participant specifically mentioned being affected by Trichotemnomania. Five participants (2.6%) exhibited Trichocryptomania (breaking off split or dry ends), and nine participants (4.6%) engaged in Trichophagy (eating hair).

More than half of the participants (54.3%) did not engage in any other BFRBDs. Of the 45.7% who did, the most common behaviors reported were skin picking (23.6%), nail biting (17.4%), and cheek biting (10.8%). Fewer participants reported lip biting (3), tongue biting (1), finger snapping (1), and nail tearing (1). Within this group, 29.2% engaged in additional BFRBDs daily, 9.2% weekly, and 4.6% less often.

**Participants’ Hair Appearance**

**Among the study sample, 50.3% of participants described their current hair density as abundant, strong, thick, or very thick, full, dense, and curly.** Another 30.2% selected "normal" to describe their hair density, while 19.5% characterized their hair as sparse, fine, thin, very thin, or bald.

**Regarding hair length, 39.5% reported long to very long hair that extends past their shoulders.** An additional 33.9% described their hair as medium-length, falling between the shoulders and the chin. **Meanwhile, 24.5% reported very short to short hair or being bald, and 4.6% reported hair of varying lengths. Regarding** hair damage or loss, 75% of participants reported areas of regrowth with hair shorter than the surrounding hair, while 53.9% noted overall hair thinning due to hair pulling. Additionally, 51.8% identified areas with no hair, and 27.2% described regions on the scalp where hair growth had permanently stopped.

Regarding hairstyle choices, **49.2% of participants wore their hair in a bun or ponytail; for example, one participant noted, "I put my hair up, and it helps me not to pull."** Other participants wore their hair loose (39.5%), pinned up (16.9%), covered (14.4%), with wigs (8.7%), braided (5.1%), or with caps (2%). **Hair descriptions indicated a noticeable perceived decline in hair quality and density after the onset (see Table S2).**

**Table S2**

| Hair Quality | Before Onset (%) | After Onset (%) |
| --- | --- | --- |
| Positive Descriptors |  |  |
| Strong, voluminous | 40.5 | 12.8 |
| Robust, thick | 35.9 | 13.8 |
| Shiny, smooth | 23.1 | 7.2 |
| Silky, soft | 10.3 | 2.6 |
| Negative Descriptors |  |  |
| Fine | 21.5 | 24.1 |
| Thin | 13.8 | 35.9 |
| Sparse | 3.6 | 27.2 |
| Dry | 16.4 | 30.8 |
| Split, broken ends | 15.4 | 26.2 |
| Frizzy, wiry | 14.9 | 22.6 |
| Scruffy | 6.7 | 19.5 |
| Oily | 7.7 | 18.5 |
| Uneven | 0.0 | 1.5 |

*Participant Describing Their Hair Before and After the Onset*
